# A Text-Book of Alkaloidal Therapeutics

**Published:** 1911-10

**Authors:** 


A TEXT-BOOK OF ALKALOIDAL THERAPEUTICS.—Being a condensed resume of all available literature on the subject of the active principles, added to the personal experience of the authors. By W. F. Waugh, M. D., and W. C. Abbott, M. D., Third edition, revised and enlarged. Chicago, 1911. Abbott Press.

"This book is dedicated to those who believe in the smallest possible quantity of the best obtainable means to produce a desired therapeutic result."

The use of crude drugs belongs to a past age. Common sense would dictate that only the active principle should be used. I remember when pulverized cinchona bark was given in hot water—a teacupful—before the days of quinine. The wonder is that we have been so long coming to our senses on the subject. This is a timely and valuable book. D.

